# Prevention and treatment of ventilator-associated pneumonia in COVID-19

**DOI:** 10.3389/fphar.2022.945892

**Published:** 2022-10-19

**Authors:** Jiayi Deng, Fanglin Li, Ningjie Zhang, Yanjun Zhong

**Affiliations:** ^1^ Department of Critical Care Medicine, The Second Xiangya Hospital, Central South University, Changsha, China; ^2^ Department of Hematology, The Third Xiangya Hospital, Central South University, Changsha, China; ^3^ Department of Blood Transfusion, The Second Xiangya Hospital, Central South University, Changsha, China

**Keywords:** ventilator-associated pneumonia, COVID-19, SARS-CoV-2, nosocomial infection, antibiotic, acute respiratory distress syndrome, ARDS

## Abstract

Ventilator-associated pneumonia (VAP) is the most common acquired infection in the intensive care unit. Recent studies showed that the critical COVID-19 patients with invasive mechanical ventilation have a high risk of developing VAP, which result in a worse outcome and an increasing economic burden. With the development of critical care medicine, the morbidity and mortality of VAP remains high. Especially since the outbreak of COVID-19, the healthcare system is facing unprecedented challenges. Therefore, many efforts have been made in effective prevention, early diagnosis, and early treatment of VAP. This review focuses on the treatment and prevention drugs of VAP in COVID-19 patients. In general, prevention is more important than treatment for VAP. Prevention of VAP is based on minimizing exposure to mechanical ventilation and encouraging early release. There is little difference in drug prophylaxis from non-COVID-19. In term of treatment of VAP, empirical antibiotics is the main treatment, special attention should be paid to the antimicrobial spectrum and duration of antibiotics because of the existence of drug-resistant bacteria. Further studies with well-designed and large sample size were needed to demonstrate the prevention and treatment of ventilator-associated pneumonia in COVID-19 based on the specificity of COVID-19.

## Introduction

Ventilator-associated pneumonia (VAP) is usually regarded as a pneumonia phenomenon that occurs within 48 h after mechanical ventilation to 48 h after extubation, and is the main type of hospital-acquired pneumonia. The risk of VAP in patients with invasive mechanical ventilation is approximately 5%–40%, and has been reported to be different depending on the country, the type of intensive care unit (ICU), and the criteria of VAP identification (American Thoracic and Infectious Diseases Society of, 2005; Seguin et al., 2014). Not only does VAP have a significant attributable mortality rate (4.6%), and VAP remains an integral part of the spectrum of adverse events such as acute respiratory distress syndrome (ARDS) (Spalding et al., 2017). VAP may place a longer hospital course and a greater financial burden on patients (Kalil et al., 2016), with a VAP-related cost of $40,144 (95% CI $36,286–44,220) reported in a survey in the United States in 2013 (Zimlichman et al., 2013). Although our knowledge of VAP has increased, its incidence has not decreased (Wang et al., 2014). Since the outbreak of COVID-19, Earth shaking changes have taken place in global medical care. COVID-19 patients had a high incidence of severe ARDS, many of whom require invasive ventilation and are at a high risk of VAP (Razazi et al., 2020; Maes et al., 2021; Fumagalli et al., 2022; Gosangi et al., 2022), which can make the state of COVID-19 patients more complicated and more strenuous to manage. Besides, COVID-19-associated VAP shows novel characters compared to non-COVID-19 disease (Wicky et al., 2021). Thus, a literature search on the PubMed and Web of Science was done for this review, using the following keywords from 2005 to 2022: “ventilator associated pneumonia” or “ventilator-associated pneumonia” or “ventilator-acquired pneumonia” or “VAP”, “COVID-19” or “SARS-CoV-2”. We aim to review the characteristics, diagnosis, drug prevention and therapy for the management of VAP in COVID-19 patients.

## Ventilator-associated pneumonia in patients with COVID-19

During the COVID-19 pandemic, severe COVID-19 patients were admitted to ICUs, received mechanical ventilation (Chang et al., 2021; COVID-ICU Group on behalf of the REVA Network and the COVID-ICU Investigators, 2021), and have a high incidence of VAP (Ippolito et al., 2021; Wicky et al., 2022).

### Occurrence and outcome of ventilator-associated pneumonia in COVID-19 patients

VAP is thought to be the most common fatal nosocomial infections in ICU (Kalanuria et al., 2014; Haque et al., 2018; Papazian et al., 2020). The reported incidence of VAP varies widely in COVID-19 patients, but was different from studies. We summarized these studies in Table 1. Some studies have shown a higher VAP risk in COVID-19 ARDS patients than in non-COVID-19 ARDS patients (Maes et al., 2021; Nseir et al., 2021; Rouzé et al., 2021; Vacheron et al., 2022a). It has been reported that VAP may bring poor prognosis to patients, with prolonged durations of both mechanical ventilation and ICU stay, and even a high mortality (Papazian et al., 2020). Similarly, the association of VAP with some relevant poor outcomes was also reported in COVID-19 patients in recent studies. Compared with non-COVID-19 patients, VAP in COVID-19 cases was significantly related with 28-day mortality, shock, VAP recurrence, and polymicrobial culture, as well as a significantly longer mechanical ventilation time (Razazi et al., 2020; Maes et al., 2021; Nseir et al., 2021; Rouyer et al., 2021). Meanwhile, compared with the influenza or non-virus infection group, no significant difference in the relationship between VAP with mortality was found in the COVID-19 group (Nseir et al., 2021).

**TABLE 1:** The characteristics of studies reported ventilator-associated pneumonia in COVID-19.

Reference no.	Sample size	VAP incidence in COVID-19 patients (VAP no./total patients no.)	VAP incidence in non-COVID-19 patients (VAP no./total patients no.)	VAP-associated outcomes
Maes et al. (2021)	225	28/1000 ventilator days (an incidence density)	13/1000 ventilator days	NA
Nseir et al. (2021)	1576	late-onset VAP: 169/200	Influenza: 79/102	1) VAP was associated with a higher risk for 28-day mortality
			No viral infection: 57/87	2) VAP was significantly associated with longer duration of mechanical ventilation
Rouyer et al. (2021)	288	42/100	188/188	1)VAP had a significantly higher rate of shock
				2) a higher rate of death in ICU
				3) a higher rate of VAP recurrence
				4) a higher risk of positive blood culture
				5) a higher rate of polymicrobial culture
				6) a higher rate of clinical worsening at day 3 and 7
Rouzé et al. (2021)	1576	205/568	Influenza:107/482	NA
			No viral infection: 87/526	
Razazi et al. (2020)	172	58/90	36/82	1) longer duration of mechanical ventilation
Vacheron et al. (2022a)	3758	550/1879	242/1879	NA
Vacheron et al. (2022b)	9129	623/1687	995/7442	1) VAP attributable mortality was higher for COVID-19 patients
				2) less likely to be extubated after a VAP

Abbreviations: VAP, ventilator-associated pneumonia; COVID-19, Coronavirus disease 2019; NA, not available.

### Risk factors for ventilator-associated pneumonia in COVID-19 patients

Potential causes for the increased incidence of VAP include prolonged mechanical ventilation and hospital stay, viral immunomodulation, steroids use, sedating and neuromuscular blocking agents use, vasopressor use, ARDS, prone positioning, applications of extracorporeal mechanical oxygenation, shortages of healthcare workers and inadequate protective equipment corresponding to the increased medical resource demands (Fumagalli et al., 2022). The number of patients requiring invasive ventilation has significantly increased during the COVID-19 pandemic, which has overwhelmed the medical resources (Nacoti et al., 2021).

The prolonged mechanical ventilation was thought to be the most relevant risk factor of VAP (Kalil and Cawcutt, 2022). The patients treated with invasive mechanical ventilation who were accompanied with COVID-19 tend to present a delayed mechanical ventilation and a higher incidence of ARDS than without COVID-19, which are both recognized as risk factors for VAP (Razazi et al., 2020; Blonz et al., 2021; Nseir et al., 2021; Rouzé et al., 2021). It has been suggested that in COVID-19 patients the use of immunomodulatory drugs may promote the development of VAP (Martínez-Martínez et al., 2021). What is more, there are also studies indicating that dexamethasone could accelerate the process of the VAP occurrence (Cour et al., 2021; Gragueb-Chatti et al., 2021). In addition, the prone position, widely applied in the treatment of COVID-19, is associated with an elevated risk of microaspiration (Ayzac et al., 2016), thereby leading to the occurrence of VAP.

### Microorganisms responsible for ventilator-associated pneumonia

The kind of microorganisms responsible for VAP reported is various, which may be explained by the duration of invasive mechanical ventilation, length of hospital and ICU stays before VAP episode, the local ecology, and cumulative exposure and timing to antimicrobials (Papazian et al., 2020). Some studies suggested that gram-negative bacteria were the predominant microorganisms (primarily *Pseudomonas aerginosa*, *Enterobacter* spp., *Klebsiella* spp.) followed by gram-positives (mainly *Staphylococcus aureus*) responsible for VAP in patients with COVID-19 (Grasselli et al., 2021; Rouzé et al., 2021). Furthermore, there is an increased occurrence of multidrug-resistant (MDR) bacterial strains in patients with COVID-19, one of the reasons may be that most cases of COVID-19-related VAP were diagnosed when invasive mechanical ventilation had been initiated for more than 7 days (late VAP) (Fumagalli et al., 2022). It is reported that multiple drug resistance of *Klebsiella pneumoniae* and *Pseudomonas aeruginosa* were isolated from COVID-19 patients (Gregorova et al., 2020; Baba et al., 2021; Ghanizadeh et al., 2021). In addition, a monocenter retrospective study involving 172 patients suggested that compared with non-COVID-19 patients, COVID-19 cases were more likely to develop MDR-related VAP (Razazi et al., 2020). Besides, COVID-19 could lead to severe organ dysfunction and prone to secondary bacterial infections, and then even facilitate the emergence of infectious diseases caused by rare microorganisms. There are also studies of VAP contributed by the rare bacteria Hafnia alvei (Cutuli et al., 2021; Méndez et al., 2021).

It is worth noting that bacteria are not the only cause of VAP. Secondary causes of fungal infection have been reported from first noticed in China, to become a clear manifestation as indicated in European in severe COVID-19 patients (Marr et al., 2021). A clinical study showed that all 134/134 (100%) patients with VAP caused by fungal were already receiving the treatment of corticosteroids and tocilizumab (Meawed et al., 2021). Current studies have shown a higher prevalence of Aspergillus pneumoniae in critical COVID-19 patients compared with the other patients in ICUs. In addition, patients affected by COVID-19 associated invasive pulmonary aspergillosis have a high mortality rate (Lahmer et al., 2021). Moreover, COVID-19 patients can also develop virus-related VAP. Herpesvirade (HSV) activation was also numerically more frequent among COVID-19 than non-COVID-19 cases (Maes et al., 2021). HSV-1 reactivation was related to the increased risks of VAP and mortality in critical COVID-19 patients (Meyer et al., 2021).

## Diagnosis of ventilator-associated pneumonia in COVID-19 patients

Diagnosing VAP in patients with COVID-19 is an appreciable challenge, since substantial overlap exists between the basic clinical symptoms and signs of COVID-19 with secondary infection (Fumagalli et al., 2022). It is considered traditionally that the diagnosis of VAP should be based on three criteria followed: clinical suspicion, new or progressive radiographic infiltrates, and microbiological diagnosis meaning positive microbiological cultures from the lower respiratory tract (Papazian et al., 2020).

### Adjustment of diagnosis for ventilator-associated pneumonia in COVID-19 patients

We should realize that it is time to consider whether there exists VAP when new clinical signs of respiratory deterioration potentially attributed by infection appear (Papazian et al., 2020). The clinical presentation of COVID-19 includes high fever, severe hypoxemia, hyperleukocytosis, biological inflammatory syndrome, and extensive bilateral radiologic infiltrates in detail. These symptoms overlap highly with VAP, making traditionally clinic diagnosis criteria for VAP invalid in the critical population with COVID-19 (Francois et al., 2020). While discovering new infiltrations in patients based on baseline infiltrations is arduous, the conventional assessment of VAP, chest X-ray or computed tomography imaging, are not suitable for real-time measurement in critical patients. Hence, lung ultrasound has been widely used as a real-time monitoring tool to monitor VAP for critically ill patients in recent years (Kameda et al., 2001; Dargent et al., 2020; Dargent et al., 2021). It must be stated that confirmed diagnosis of VAP depends on the identification of the pathogen. Regardless of the technique used (endotracheal aspiration or bronchoscopy-guided bronchoalveolar lavage), a challenge in diagnosing VAP is reducing the time from sampling to pathogen identification (Papazian et al., 2020).

The widespread use of new molecular technology effectively alleviates this problem and contributes to the increasing incidence of VAP. Tools based on multiplex polymerase chain reaction make it possible to diagnose early VAP and identify VAP usually underdiagnosed by conventional culture-based methods (Cohen et al., 2021; Wicky et al., 2022). It has been reported in the literature, the combination of sequential PCR and electrospray ionization mass spectrometry was a potential rapid technique to diagnose VAP within 6 h (Hou et al., 2020). In addition, the next-generation sequencing (NGS) and even metagenomic NGS (mNGS) are in some distance more effective and rapid for pathogen detections (Toma et al., 2014; He et al., 2022). NGS-based methods and mNGS-based methods can help clinicians to make accurate and precise diagnosis, leading to targeted antimicrobial therapy and improve the prognosis of patients in time.

### Other methods to help identify ventilator-associated pneumonia in COVID-19 patients

It is reported that procalcitonin (PCT) can help distinguish virus from bacterial pathogens in patients with VAP, and typical bacteria tend to present higher procalcitonin levels than atypical bacteria or viruses (Self et al., 2017; Modi and Kovacs, 2020). The data of a clinical study suggested that the patients whose PCT was over 0.975 ng/ml were more likely to have VAP, showing that PCT may be an applicable biomarker for VAP diagnosis (Côrtes et al., 2021). In addition, immature granulocytes (IGs) was considered that the threshold was 18% or 2 g/L, and the sensitivity and specificity to identify patients with ventilator-associated pneumonia were 100%, supporting IGs could be a biomarker to help identify pulmonary bacterial infections in this population (Daix et al., 2021).

## Drug treatment of ventilator-associated pneumonia in COVID-19 patients

### Antimicrobial therapy

Intravenous antimicrobial therapy is the cornerstone for drug treatment of VAP. It must be stressed that the emergence of antimicrobial resistance and determining the appropriate type, timing and duration of antibiotics is worthy of attention.

COVID-19 patients on mechanical ventilation have a very high risk of exposure to extensive antibiotic treatment. During their hospitalization, most patients were prescribed several different classes of antibiotics, of which broad-spectrum coverage was commonly used. Cefepime and vancomycin have been reported to be the most commonly used antibiotics, with an average duration of 1 week. (Risa et al., 2021). It is generally recommended that the empirical treatment plan should be based on the local distribution of VAP-related pathogens and their antimicrobial susceptibility, and resistance rates vary widely among countries, regions, and hospitals. Guideline-based empirical antibiotic management results in antibiotic overuse (Pickens et al., 2021). Inappropriate empiric antibiotic use may lead to the emergence of more resistant bacteria.

Antimicrobial resistance is not only a global crisis but also a global problem that attracts the attention of governments and society. Multiple studies showed that drug-resistant and even multidrug-resistant frequently occurred in COVID-19 patients with VAP. Since the outbreak of COVID-19, antibiotic resistance is increasing. The resistance to the ceftazidime and levofloxacin for *P. aeruginosa* strains was significantly increased, as well as A. baumannii strains (Bahçe et al., 2022). Therefore, drugs for multidrug resistant bacteria infection of VAP, such as polymyxins, ceftazidime avibatan et al. were used to improve the outcomes of COVID-19 patients. Moreover, recent researches suggested that PBT2 may act as a drug resistance inhibitor to rescue the efficacy of commonly used tetracycline antibiotics in the treatment of multidrug-resistant baumannii infection (De Oliveira and Walker, 2022).

The best way to determine a specific antibiotic depends on the evidence of pathogen culture from bronchoalveolar lavage or endotracheal aspiration of the lower airways (Fumagalli et al., 2022). New antimicrobials are undergoing rapid development, aiming to keep pace with the development of multidrug resistance (Cusack et al., 2022). We expect more target drugs to be developed earlier in the future, to implement more precise etiological treatment.

### Other drugs treatments

An observational study found that it may be a reasonable therapeutic option to decrease the intubation rate in COVID-19 patients (Mushtaq et al., 2022). Compared with high-dose dexamethasone, tocilizumab seemed to be a much better and safer for controlling the cytokine storm in COVID-19 patients with moderate to severe ARDS (Naik et al., 2021). In contrast to this study, tocilizumab was reported to increase the incidence of VAP in critical COVID-19 patients (Ceccarelli et al., 2021). In addition, it is reported that combination therapy of tocilizumab and steroids is likely to be conducive to managing COVID-19-associated cytokine release syndrome (Dravid et al., 2021). While another research suggested that adding tocilizumab to methylprednisolone did not improve outcomes significantly (Hamed et al., 2021). Further, interferon gamma is proved to have a plausible efficacy in the treatment of recurrent VAP by recovering monocyte activation (Nguyen et al., 2021).

## Drug prevention of ventilator-associated pneumonia in COVID-19 patients

VAP is difficult to manage and it will complicate existing diseases, so we should pay attention to its prevention which is thought to be more important than the treatment of VAP. It is generally believed that the main way to prevent VAP is to reduce the timing of invasive mechanical ventilation, and this part focuses on the pharmacological prevention of VAP.

### Stress ulcer prophylaxis

Ventilated patients usually need to use proton pump inhibitors (PPI) and gastric mucosal protective agent prophylactically because of the risk of stress ulcers. These acid suppressive medications inhibit gastric acid secretion, increase the hydrogen of the gastric juice, and promote bacterial growth (Buendgens et al., 2016). It may be a reason of stress ulcer prophylaxis was reported to be associated with higher VAP rates (Alhazzani et al., 2018; Huang et al., 2018). There were a lot of researches focused on the association of PPI use with COVID-19 (Almario et al., 2020; Elmunzer et al., 2021; Fan et al., 2021; Lee et al., 2021; Ramachandran et al., 2022), and the results showed that PPI use was associated with the increased susceptibility of COVID-19 infection and poor outcome including disease severity and mortality (Fatima et al., 2022). However, the relationship between PPI use and VAP in COVID-19 patients still needs to be demonstrated. A meta-analysis suggested that sucralfate, a gastric mucosal protective agent, could significantly decrease the occurrence of VAP, but cannot affect the days on ventilator, duration of ICU stay, and ICU mortality (He et al., 2014). Hence, sucralfate would be a good choice to prevent stress ulcers in critical ill patients with invasive mechanical ventilation.

### Selective digestive decontamination

Selective digestive decontamination (SDD) is demonstrated to be effective in reducing the occurrence of VAP in non-COVID-19 patients (Liberati et al., 2009; Minozzi et al., 2021; van der Meer et al., 2021). In COVID-19 patients, a retrospective observational study that included 178 subjects on invasive mechanical ventilation more than 2 days, showed that the use of SDD significantly reduced the incidence of VAP (Luque-Paz et al., 2022). But, the evidence level of this study limited the reliability for its retrospective and observational design. Therefore, SDD deserves more consideration to use in critical COVID-19 patients.

### Chlorhexidine

Chlorhexidine, a drug used in oral hygiene care for over 2 decades, can reduce oral colonization, and prevent the occurrence of VAP (Zand et al., 2017). Besides, the combination of chlorhexidine with toothbrushing was more satisfactory in preventing VAP in patients on mechanical ventilation, compared with chlorhexidine alone (Silva et al., 2021). However, several studies were questioning the efficacy and safety of oral chlorhexidine. It has been reported in some studies that the use of chlorhexidine oral care may increase mortality, owing to the occurrence of acute lung injury resulting from aspirating the anticorrosive composition of chlorhexidine (Klompas et al., 2014a; Klompas, 2017; Deschepper et al., 2018; Harris et al., 2018). In COVID-19 patients, Chlorhexidine was reported to be effective in reducing SARS-CoV-2 load in the oral cavity (Costa et al., 2021). More research is needed in the future to clarify the safety and efficacy of chlorhexidine in preventing VAP for COVID-19 patients.

### Prophylactic probiotics

The microbiota plays an important role in the risk of intestinal complications and the disease severity of COVID-19 patients (Zanza et al., 2021). Probiotics may be an attractive intervention for preventing VAP in adult hospitalized patients by modulating the intestinal microbiome and reducing the colonization of pathogens (Papazian et al., 2020; Su et al., 2020; Kullar et al., 2021). Systematic reviews also support the protective role of probiotics in preventing VAP (Rozga et al., 2021). But the evidence levels of these included studies were low and existing significant heterogeneity, the use of probiotics for preventing VAP in COVID-19 patients remains controversial.

### Early enteral nutrition

Early enteral nutrition is recommended to prevent VAP in critically ill non-COVID-19 patients (Klompas et al., 2014b) and critical COVID-19 patients (Haines et al., 2022). A study of real-world clinical practice showed that early enteral nutrition within 3 days after invasive mechanical ventilation can shorten the time of invasive mechanical ventilation and improve the outcome of COVID-19 patients (Haines et al., 2022). The mechanisms of early enteral nutrition in preventing VAP may involve reducing pathogen colonization and bacterial translocation through facilitating intestinal peristalsis and maintaining intestinal mucosal structure and barrier function. However, a large number of patients failed to initiate early enteral nutrition for hemodynamic instability, fear of aspiration, and significant gastrointestinal complications.

### Intravenous selenium

A narrative literature review proved that selenium supplementation could reduce the incidence of VAP, shorten the length of hospital stay, and decrease mortality through decreasing the inflammatory cytokines (Mahmoodpoor et al., 2018; Oliveira et al., 2022). In COVID-19 patients, a higher prevalence of selenium deficiencies was found, especially in older cases (Voelkle et al., 2022). However, direct evidence of selenium supplementation in preventing VAP is needed in further research.

## Conclusion

As mentioned above, during the COVID-19 era, VAP has drawn more attention than before due to its high incidence and high mortality. Patients who develop ARDS and require invasive mechanical ventilation after SARS-CoV-2 infection are at a higher risk to experience VAP episodes than non-COVID-19 ARDS patients. Despite extensive research, the diagnosis, prevention and treatment of VAP remain a challenge. At present, strict hospital management measures and standardized procedures to prevent VAP. As for clinical treatment, the application of antibiotics remains recommended, especially the resistance of antibacterial drugs is a serious problem, attracting people’s attention. Further studies with well-designed and large sample size were needed to demonstrate the prevention and treatment of ventilator-associated pneumonia in COVID-19 based on the specificity of COVID-19.
